# Determination of the relationship between mothers’ carbohydrate counting knowledge and glycemic control in children with type 1 diabetes

**DOI:** 10.1007/s00508-025-02696-3

**Published:** 2026-01-23

**Authors:** Sercan Özbek Yazici, Beyza İlişiksiz, Sümeyye Kemaneci, Mustafa Özgür Pirgon

**Affiliations:** 1 Health Sciences Faculty, Department of Nutrition and Dietetics, Burdur Mehmet Akif Ersoy University, Burdur, Turkey; 2 Department of Child Health and Diseases, Pediatric Endocrine, Suleyman Demirel University Medicine Faculty, Isparta, Turkey

**Keywords:** Carbohydrate counting, Children, Diabetes mellitus, Education level, Glysemic control

## Abstract

**Background:**

Carbohydrate counting (CC) supports glycemic control. In children with type 1 diabetes mellitus (T1DM), mothers are usually the primary caregivers and parental knowledge and involvement play a key role in diabetes management.

**Objective:**

This study aims to evaluate mothers’ knowledge of CC and the impact of their knowledge level on glycemic control (glycated hemoglobin, HbA1c) in children and adolescents with T1DM.

**Methods:**

The research was an analytical cross-sectional study conducted at the Pediatric Endocrinology Polyclinic. This study was conducted with 102 mothers of children and adolescents with T1DM. Mothers’ CC knowledge was assessed using a test designed by the researchers. All participants completed demographic and clinical questionnaires. The measurement of HbA1c was used to evaluate glycemic control, where HbA1c levels < 7.5% represented good control and ≥ 7.5% indicated poor control. Multiple linear regression analysis was performed to evaluate the determinants of HbA1c (%).

**Results:**

The mean HbA1c level of children and adolescents was 8.3% ± 1.87, with only 35.3% achieving good glycemic control. The study demonstrated that mothers possess a certain level of CC knowledge. Children and adolescents of mothers who had received a university education had lower HbA1c levels (*p* < 0.05). There was no significant relationship between mothers’ education levels and their CC knowledge score. The HbA1c levels showed a negative correlation with mothers’ CC knowledge score (r:−0.315). Mothers of children with good glycemic control had a significantly higher CC knowledge score (*p* < 0.05). The CC knowledge score of mothers had a negative correlation with HbA1c (%). In multiple regression analyses mothers’ education levels (B:−0.318, *p* < 0.05) and CC knowledge score (B:−0.177, *p* < 0.05) were significant predictors of HbA1c, explaining 20.8% of the variance.

**Conclusion:**

The findings suggest that there is a need to enhance mothers’ CC knowledge. Mothers’ CC knowledge levels were found to affect their children’s glycemic control. Therefore, regular training should be provided to improve healthy eating habits and accurate carbohydrate counting knowledge, and mothers’ knowledge levels should be assessed to address any gaps.

**Supplementary Information:**

The online version of this article (10.1007/s00508-025-02696-3) contains supplementary material, which is available to authorized users.

## Introduction

Type 1 diabetes mellitus (T1DM) is the second most common chronic condition affecting children and is characterized by insulin deficiency and hyperglycemia [1]. In patients with T1DM inadequate glycemic control can lead to macrovascular and microvascular complications in the long term; therefore, effective management of T1DM is of great importance [2]. Individuals with T1DM need appropriate daily insulin treatment, regular blood glucose monitoring, physical activity, healthy nutrition, education and support to delay or prevent diabetes-related complications [3]. Especially in children and adolescents with T1DM, medical nutrition treatment that supports glycemic control along with optimal growth and development is a fundamental component of diabetes management [4].

Carbohydrates in a meal are a significant nutritional determinant of postprandial glucose levels, glycemic variability and glycated hemoglobin levels (HbA1c, %) [5]. Therefore, one of the main components of T1DM management is carbohydrate counting (CC) to determine prandial insulin doses [6]. The use of CC is a meal planning approach [5] and good glycemic control is one of the significant benefits [6]. It has been found to support optimal metabolic control in pediatric and adolescent patients with T1DM, without increasing weight gain or insulin requirements. Therefore, the accuracy of knowledge and proper use of CC by individuals with diabetes and their families is critically important for its effectiveness [4].

In pediatric individuals with T1DM, the primary caregiver is most often the mother. Parental knowledge and education regarding T1DM and nutritional management, along with their active involvement in the child’s diabetes care, are important tools for achieving desired outcomes in diabetes management [7, 8]. Periodic assessments can identify and address knowledge gaps [7]. Waheed et al. (2023) highlighted that tests assessing carbohydrate knowledge levels can help identify knowledge gaps and provide educational opportunities that may improve glycemic outcomes in individuals with T1DM [6].

This study aims to evaluate mothers’ CC knowledge and the impact of their knowledge level on glycemic control (HbA1c %) in children and adolescents with T1DM. Additionally. The effects of demographic and clinical characteristics on HbA1c levels were assessed.

## Methods

### Study setting and design

This analytical cross-sectional study was conducted at Suleyman Demirel University Research and Education Hospital in Isparta, Turkey, between 15 November 2024 and 15 February 2025.

The study population consisted of mothers of children and adolescents who were diagnosed with T1DM and regularly followed up at the Pediatric Endocrinology Polyclinic, Suleyman Demirel University Research and Education Hospital. Without using sample selection methods, the study included mothers of children and adolescents with T1DM who attended regular follow-ups during the study period. The sample of the study was calculated using the results of a similar study conducted previously [7] using the GPower 3.1 Package Program (Kiel University, Germany) with a type 1 error level of α = 0.05 and power 1 − β = 0.95. The minimum sample size was found to be 46 people and with a 10% reserve, this number was determined as 51.

The study included mothers of children aged 18 years or younger who had been diagnosed with T1DM for at least 6 months. Eligible participants were those whose child had been using an insulin pump or insulin pen for at least 3 months and who had sufficient reading and comprehension skills. Those who did not meet the inclusion criteria were excluded from the study. In this context, the study was conducted with the mothers of 102 children and adolescents (56 female, 46 male). The aim was to reach mothers of female and male children as equally as possible.

### Data collection

Data were collected through the completion of questionnaires by mothers of children and adolescents with T1DM who applied to the outpatient clinic.

In the evaluation of individual characteristics, a questionnaire was used that included information on the family (mother and father’s education level) and general information about the child or adolescent with T1DM (age, sex, physical activity status), clinical characteristics, e.g., time since diagnosis, most recent measured HbA1c levels (%), CC training and CC use, insulin regimen (pen or pump), insulin dose (bolus and basal), glucose monitoring method (self-monitoring blood glucose, SMBG; SMBG using finger-pricking) or continuous glucose monitoring (CGM) sensor.

Mothers’ CC knowledge was assessed using a test. The use of CC is defined at three levels in national guidelines: basic, intermediate and advanced [9, 10]. The basic level aims to teach about carbohydrate-containing foods, the intermediate level aims to evaluate the relationship between food consumption and blood glucose and the advanced level aims to determine the short-acting or rapid-acting insulin dose to be administered based on carbohydrate intake and blood glucose levels [10]. In this study, CC knowledge test contains 17 questions related to CC, prepared by researchers and categorized into 3 levels: basic (5 questions), intermediate (9 questions) and advanced (3 questions). Of the questions, 10 are true and 7 are false propositions (Supplementary file). Each question was scored as “1” for a correct answer and “0” for an incorrect answer [8, 9]. Scores from each level and the total score from all questions were summed to obtain level-specific and overall knowledge scores. The estimated time for completing the quiz was 10–15 min.

At the outset of the study, participating mothers received comprehensive information about the research objectives and their written consent was obtained. Ethical approval was obtained from the Institutional Ethics Committee.

### Evaluation of glycemic control

Glycemic control levels of children and adolescents were assessed using glycated hemoglobin (HbA1c, %). Good glycemic control was defined as HbA1c levels below 7.5%, whereas poor control was defined as levels of 7.5% or higher [1, 11, 12].

### Statistical analysis

The data were analyzed using the SPSS statistical software (IBM, Armonk, NY, USA). Descriptive statistics are presented as mean (X) ± standard deviation (SD) for variables with normal distribution, median and interquartile range (IQR) values for variables with non-normal distribution and normal variables as number of cases and percentage (%). To compare qualitative variables Student’s t‑test was used when normal distribution assumptions were met and the Mann-Whitney U test was used otherwise. The χ^2^-test was applied to examine relationships between categorical variables. For the relationship between two numerical variables, Pearson’s correlation coefficient was used if the data were normally distributed and if not, Spearman’s correlation coefficient was used. Additionally, multiple linear regression was performed to model the relationship between multiple independent variables and a single dependent variable. To evaluate the effect of a qualitative variable with three or more categories on a numerical variable, the one-way ANOVA test was used when the distribution was normal and the Kruskal-Wallis test was used when it was not. A 95% confidence level was used for all analyses and results were considered statistically significant at *p* < 0.05.

## Results

This study was conducted with mothers of children and adolescents with T1DM. A total of 102 mothers were included in the study. Information regarding the children and adolescents with T1DM was obtained from the mothers.

Tables 1 and 2 present the demographic and clinical characteristics of the mothers and the children and adolescents with T1DM. The age of children and adolescents with T1DM ranged from 2–17 years and 54.9% were female. The fathers of 46.1% and the mothers of 38.2% of the children and adolescents were university graduates. It was determined that approximately 1 in 4 (24.5%) children have a family history of diabetes and 47.1% of children did not engage in physical activity. Regarding insulin treatment methods, 22.5% of the children and adolescents were using an insulin pump, while 58.8% were monitoring the blood glucose levels with a CGM device. The proportion of those who received CC training was 70.6%, while 58.8% actively used it. A statistically significant relationship was found between the mother’s education level and the child’s HbA1c (*p* < 0.05), although no significant relationship was found between the father’s education level and HbA1c (*p* > 0.05). It was found that sex, family history of diabetes and physical activity status had no significant effect on glycemic control (HbA1c, %) (Table 1).

**Table 1:** Comparison of children and adoscents’ HbA1c level and mothers’ CC knowledge score with demographic and clinical characteristics

	*N*	%	HbA1c (%) X ± SD	CC knowledge total score X ± SD
**Sex (children)**				
Female	56	54.9	8.4 ± 1.86	10.6 ± 2.39
Male	46	45.1	8.2 ± 1.81	10.7 ± 2.46
*p*	–	–	0.602^c^	0.832^c^
**Mother educational level**				
Primary school	19	18.6	9.0 ± 2.36	10.3 ± 1.85
Middle school	15	14.7	8.6 ± 1.44	9.9 ± 2.43
High school	29	28.4	8.5 ± 2.1	10.5 ± 2.87
University	39	38.2	7.6 ± 1.83	11.2 ± 2.25
*p*	–	–	**0.013**^**b**^	0.309^d^
**Father educational level**				
Primary school	6	5.9	9.5 ± 1.79	10.0 ± 1.26
Middle school	11	10.8	8.7 ± 2.25	10.1 ± 2.16
High school	38	37.3	8.5 ± 1.68	10.4 ± 2.78
University	47	46.1	7.8 ± 1.79	11.0 ± 2.25
*p*	–	–	0.085^d^	0.496^d^
**Family history of diabetes**				
Yes	25	24.5	7.9 ± 1.42	9.9 ± 2.84
No	77	75.5	8.4 ± 1.94	10.8 ± 2.23
*p*	–	–	0.235^c^	0.064^c^
**Physical activity status**				
Yes	54	52.9	8.4 ± 1.84	10.9 ± 2.24
No	48	47.1	8.1 ± 1.83	10.3 ± 2.58
*p*	–	–	0.450^c^	0.231^c^
**Insulin treatment**				
Pump	23	22.5	8.0 ± 1.21	10.4 ± 2.69
Pen	79	77.5	8.4 ± 1.98	10.7 ± 2.34
*p*	–	–	0.440^c^	0.612^c^
**Glucose monitoring method**				
SMBG	42	41.2	8.6 ± 2.08	10.2 ± 2.45
CGM	60	58.8	8.1 ± 1.62	10.9 ± 2.36
*p*	–	–	0.312^a^	0.136^c^
**CC training**				
Yes	72	70.6	8.4 ± 1.96	10.7 ± 2.37
No	30	29.4	8.1 ± 1.48	10.5 ± 2.54
*p*	–	–	0.495^c^	0.686^c^
**CC use**				
Yes	60	58.8	8.2 ± 1.87	10.8 ± 2.32
No	42	41.2	8.4 ± 1.79	10.4 ± 2.55
*p*	–	–	0.608^c^	0.365^c^
**HbA1c group**				
Good control (< 7.5%)	36	35.3	6.8 ± 0.47	11.3 ± 2.53
Poor control (≥ 7.5%)	66	64.7	9.1 ± 1.77	10.2 ± 2.24
*p*	–	–	**0.000**^**a**^	**0.020**^**c**^

*CC* carbohydrate counting, *CGM* continuous glucose monitoring, *HbA1c* glycated hemoglobin, *SD* standard deviation, *SMBG* self-monitoring blood glucose, *X* mean

^a^Mann-Whitney U test, ^b^Kruskal-Wallis test, ^c^Studentʼs t test and ^d^one way ANOVA were applied

**Table 2:** Demographic and clinical characteristics of children/adolescents with T1DM and mothers’ CC knowledge scores

	X ± SD	Median (IQR)
Age of children (years)	11.2 ± 3.93	11.0 (5.00)
Time since diagnosis (years)	2.5 ± 0.98	2.0 (1.00)
B/b insulin ratio	1.4 ± 0.70	1.3 (1.00)
HbA1c (%)	8.3 ± 1.87	7.9 (1.90)
CC knowledge total score	10.5 ± 2.41	11.0 (3.00)
Basic CC knowledge score	2.9 ± 1.18	3.0 (2.00)
Intermediate CC knowledge score	5.5 ± 1.81	6.0 (3.00)
Advanced CC knowledge score	2.0 ± 0.76	2.0 (1.00)

*B/b* bolus-to-basal, *CC* carbohydrate counting, *HbA1c* glycated hemoglobin, *IQR* interquartile range, *SD* standard deviation, *X* mean

The mean age of the children and adolescents was 11.2 ± 3.93 years and the mean time since diagnosis was 2.5 ± 0.98 years. The mean HbA1c level among children and adolescents was 8.3 ± 1.87% (Table 2). The mean bolus-to-basal (B/b) insulin ratio was 1.4 ± 0.70. The total CC knowledge score of participants had a mean of 10.5 ± 2.41 (Table 2).

The study compared diabetes management characteristics between children with T1DM who had good glycemic control and those with poor glycemic control. No statistically significant differences were observed between good and poor glycemic control groups in terms of insulin treatment, glucose monitoring methods, CC training or CC use (Table 3). These variables do not appear to have a significant impact on the glycemic control status of the children.

**Table 3:** Diabetes and treatment-related characteristics according to glycemic control

Variable	Good glycemic control (< 7.5%) *N* (%)	Poor glycemic control (≥ 7.5%) *N* (%)	*p*-value*
*Insulin treatment*			
Pump	7 (10.8)	16 (15.7)	0.579
Pen	29 (28.4)	50 (49.0)	
*Glucose monitoring method*			
SMBG	14 (13.7)	28 (27.5)	0.729
CGM	22 (21.6)	38 (37.3)	
*CC training*			
Yes	27 (26.5)	45 (41.1)	0.470
No	9 (8.8)	21 (26.6)	
*CC use*			
Yes	24 (23.5)	36 (35.3)	0.235
No	12 (11.8)	30 (29.4)	

*CC* carbohydrate counting, *CGM* continuous glucose monitoring, *SMBG* self-monitoring blood glucose

*χ^2^-test was applied

Correlation analysis identified statistically significant linear relationships among diabetes-related parameters (Table 4). Both age and time since diagnosis showed a negative correlation with the B/b insulin ratio (*p* < 0.05). The HbA1c level was negatively correlated with both CC knowledge total score (r: −0.315, *p* < 0.05) and B/b ratio (r: −0.260, *p* < 0.05). Notably, no significant correlation was observed between CC knowledge score and the B/b insulin ratio (r: 0.027, *p* > 0.05).

**Table 4:** Correlation between age, time since diagnosis, HbA1c, CC knowledge total score and B/b insulin ratio

	Age (year)			Time since diagnosis (years)			HbA1c (%)			CC knowledge total score		
	r	*p*	95% CI	r	*p*	95% CI	r	*p*	95% CI	r	*p*	95% CI
Age (years)	1.000	–	–	–	–	–	–	–	–	–	–	–
Time since diagnosis (years)	0.451	0.000	0.284–0.597	1.000	–	–	–	–	–	–	–	–
HbA1c (%)	0.252	0.010	0.053–0.441	0.228	0.021	0.036–0.431	1.000	–	–	–	–	–
CC knowledge total score	−0.077	0.350	−0.287–0.090	0.049	0.677	−0.155–0.211	−0.315	0.001	−0.490–−0.124	1.000	–	–
B/b insulin ratio	−0.283	0.004	−0.476–−0.101	−0.230	0.021	−0.409–−0.053	−0.260	0.009	−0.445–−0.079	0.027	0.786	−0.167–0.214

*B/b* bolus-to-basal, *CC* carbohydrate counting, *CI* confidence interval, *HbA1c* glycated hemoglobin

Spearman correlation was applied

The results of multiple linear regression analyses evaluating the determinants of glycemic control (HbA1c %) are presented in Table 5. Mothers’ CC knowledge (B: −0.177, *p* < 0.05) and education level (B: −0.318, *p* < 0.05) were found to be significant determinants of HbA1c (%). These findings indicate that higher mothers’ CC knowledge and education levels were associated with lower HbA1C (%). The model explains 20.8% of the variance.

**Table 5:** Multiple linear regression analysis indicating the predictors of HbA1c (%)

	Nonstandardized coefficients		Standardized coefficients					95% CI		
	B	SE	β	t	Sig	F	Sig	Lower bound	Upper bound	R^2^
Constant	9.511	1.013	–	9.393	*<* *0.001*	6.368	*<* *0.001*	7.501	11.520	0.208
Age (years)	0.087	0.047	0.191	1.858	0.066	–	–	−0.006	0.180	–
Time since diagnosis (years)	0.250	0.189	0.134	1.320	0.190	–	–	−0.126	0.626	–
CC knowledge total score	−0.177	0.070	−0.234	−2.535	*0.013*	–	–	−0.316	−0.039	–
Mother educational level	−0.318	0.153	−0.195	−2.078	*0.040*	–	–	−0.621	−0.014	–

*CC* Carbohydrate counting, *CI* confidence interval, *SE* standard error, *sig* significant

## Discussion

This study offers an important perspective for family-centered diabetes management by linking mothers’ CC knowledge to children and adolescent’s glycemic outcomes, emphasizing that diabetes education should not only target patients but also empower caregivers. In the present study, mothers’ CC knowledge scores of children with good glycemic control were significantly higher than those of mothers of children with poor glycemic control and there was a statistically significant negative correlation between the CC knowledge total score and HbA1c (%) level. A 1-point increase in the CC knowledge total score was associated with a 0.177 decrease in HbA1c (%). A significant association was observed between maternal education level and HbA1c levels in children and adolescents with T1DM. Regression analysis exhibited that increasing the mother’s education level resulted in a 0.318 decrease in HbA1c (%). Consistent with previous studies, higher maternal education was associated with better glycemic control [8, 11]. Interestingly, no significant relationship was found between mothers’ education levels and their CC knowledge scores. This finding suggests that proficiency in CC is a specific skill that can be effectively taught to parents through diabetes-specific education, regardless of their formal educational background. As a result, this study determined that children of mothers with higher CC knowledge scores had lower HbA1c levels. This finding supports previous studies which highlight that greater knowledge about diabetes in mothers, including dietary aspects, is associated with better HbA1c levels in children [8, 11]; however, although better nutritional and CC knowledge have been reported to improve glycemic control [6, 13, 14], the evidence regarding the association between CC training and HbA1c levels remains conflicting. In a study by Tandon et al. (2024) [5] comparing the effects of routine nutrition education and basic CC training on glycemic control in individuals with T1DM (aged 6–25 years), it was found that basic CC training did not lead to improvement in glycemic control but it facilitated flexibility in food choices and perceived ease in insulin adjustments. Similarly, another study found that basic and advanced CC training did not provide a significant advantage over individualized diet counselling for overall glycemic control. These varying results have been explained by the necessity to consider learning preferences when determining the appropriate diet training approach for individuals with diabetes in medical nutrition treatment [15].

The study found that 70.6% of mothers received CC training, yet 58.8% actively implemented this method in daily practice. Neither CC training nor its active use was found to significantly affect children’s glycemic control. This finding shows that even when CC training is received, the expected benefit in diabetes management cannot be achieved unless it is correctly and consistently implemented. Therefore, it is crucial to periodically repeat CC training and to monitor the accuracy of CC implementation. Supporting this conclusion, a study by Marker et al. (2019) [13] found that the level of CC knowledge was negatively associated with the time since the diabetes diagnosis, indicating the need for continuous education to maintain nutritional knowledge related to diabetes over time.

The HbA1c levels of children and adolescents showed a positive correlation with the time since diagnosis and age. This finding reveals that glycemic control worsens with increasing age and the time since diagnosis. This result is consistent with other studies [2, 16] and supports the finding that blood glucose control is more difficult in adolescence [17]. Family conflicts negatively affect glycemic control in children and adolescents [17, 18]. Adolescence is a critical period of significant physical and psychological development and endocrinological changes, decreased compliance with treatment and increased susceptibility to psychological problems such as depression, anxiety and eating disorders have been associated with difficulties in managing T1DM [19]. This highlights that during this period where glycemic control becomes more challenging, the role of a knowledgeable mother becomes even more critical for successful diabetes management.

The SMBG and CGM are known to be effective tools for glycemic management. The last 20 years have witnessed substantial growth in diabetes technology use, especially of insulin pumps and CGM systems and have transformed diabetes care [20]. This trend is particularly evident in the pediatric population [21]. The present study revealed a predominance of insulin pen use (77.5%) over pump use (22.5%) while the rate of CGM usage was 58.8%. In a recent study conducted in Türkiye, Donbaloğlu et al. (2024) [22] reported that rates of pump and CGM use were 37.8% and 29.9%, respectively. Children using either insulin pumps or CGM systems had lower HbA1c levels than non-users but these differences were not statistically significant. Moreover, there was no statistically significant association of glycemic control status with either the insulin treatment method or the glucose monitoring method. Several studies showed that pump and CGM use contribute to reduced HbA1c levels [18, 22]. Other studies reported that pump use causes a small reduction in HbA1c that is not clinically significant [21] and that CGM use does not affect glycemic control [23]. The results suggest that effective glycemic control may be dependent on more factors than just diabetes technologies. In a meta-analysis it was found that the use of insulin pumps, high-frequency sensor monitoring, early use of insulin pumps, male gender and mothers’ compliance characteristics were associated with reduced HbA1c levels in children and adolescents, highlighting the impact of different variables on glycemic control [18].

Standard insulin treatment for T1DM typically involves a combination of premeal rapid-acting insulin and long-acting basal insulin. This form of treatment is known as basal-bolus therapy and reduces the risk of hypoglycemia, particularly when insulin analogues are used [24]. Recommendations currently rely on estimated basal insulin requirements, which typically represent 30–50% of the total insulin dosage [25, 26]. A study in the pediatric population found that low-dose basal insulin infusion had a positive effect on HbA1c levels [26]. Similarly, Yamada et al. (2017) [27] found that a lower basal insulin ratio (approximately 30% of the total daily dose) was associated with improved glycemic control (HbA1c ≤ 7.5%) in adults with T1DM. In the present study, a negative correlation was found between the B/b insulin ratio and HbA1c levels, supporting previous evidence that a lower proportion of basal insulin contributes to improved glycemic outcomes. González-Vidal et al. (2024) [24] indicated that a higher percentage of basal insulin (PBI) is associated with an increased tendency toward hyperglycemia, whereas a lower PBI is linked to the higher glucose variability. These findings highlight the importance of the B/b ratio as an approach to personalizing insulin treatment to improve glycemic control.

The study has some limitations. The first of the limitations is that the participants are mothers of children and adolescents with T1DM who were followed by the Süleyman Demirel University Research and Education Hospital Pediatric Endocrinology Polyclinic for a certain period. Therefore, the sample was selected nonrandomly. The second limitation of the study is that the reliability and validity of the questions evaluating the level of CC knowledge were not assessed. Another limitation of our study is that the physical activity of children and adolescents was based on mothers’ statements. The final limitation of the study is that the Time in Range (TiR) value was not available for some individuals and therefore was not included in the study.

In conclusion, the findings suggest that there is a need to enhance mothers’ knowledge of CC. Also, mothers’ CC knowledge levels and education levels particularly affect their children’s glycemic control. Given the critical role of mothers’ CC knowledge in pediatric glycemic control, structured education programs should focus on enhancing children’s self-management skills, reinforcing maternal knowledge and conducting periodic assessments to maintain optimal dietary management in T1DM. Additionally, incorporating feedback evaluation, problem-solving strategies and the development of digital tools, such as mobile applications for carbohydrate calculation, glycemic index tables and diabetic plate method, may further improve the effectiveness of these interventions.

## Supplementary Information

ESM1: Supplementary material 1
