# Supplementary material for: Determination of the relationship between mothers’ carbohydrate counting knowledge and glycemic control in children with type 1 diabetes
Source: Wien Klin Wochenschr. 2026 Jan 23;138(15-16):470–7. doi: 10.1007/s00508-025-02696-3 (PMC13493431; doi:10.1007/s00508-025-02696-3)
Supplement: Supplementary file 1 [file 508_2025_2696_MOESM1_ESM.docx]

**Determination of the Relationship Between Mothers' Carbohydrate Counting Knowledge and Glycemic Control in Children with Type 1 Diabetes**

### **Sercan ÖZBEK YAZICI*, Beyza İLİŞİKSİZ, Sümeyye KEMANECİ, Mustafa Özgür PİRGON**

*Health Sciences Faculty, Department of Nutrition and Dietetics, Burdur Mehmet Akif Ersoy University, Burdur, Turkey, sozbekyazici@hotmail.com

**CC knowledge questions**

|  |  | **True** | **False** |
| --- | --- | --- | --- |
| **Basic CC knowledge questions** | Among the nutrients, carbohydrates have the greatest impact on postprandial blood glucose levels. |  |  |
|  | The same amount of milk contains more carbohydrates than meat. |  |  |
|  | The effect of proteins on blood glucose is so **minimal** that it is considered negligible. |  |  |
|  | One food exchange of grains contains more carbohydrates than one food exchange of legumes. |  |  |
|  | One serving of fruit and one serving of bread contain the same amount of carbohydrates. |  |  |
| **Intermediate CC knowledge questions** | The glycemic index indicates the average blood glucose level over a three-month period. |  |  |
|  | Carbohydrate sources with high fiber content have a higher glycemic index. |  |  |
|  | The total amount of carbohydrates is more important than the carbohydrate source in raising blood glucose levels. |  |  |
|  | High-fat foods cause a delayed rise in blood glucose levels. |  |  |
|  | There is no harm in consuming diabetic packaged products without checking the label information. |  |  |
|  | The presence of the phrase "no added sugar" on a food label does not always indicate that the food is appropriate for consumption. |  |  |
|  | Bananas have a moderate glycemic index. |  |  |
|  | A food with a low glycemic load can be eaten in unlimited quantities without concern for the amount. |  |  |
|  | A food label stating it contains 30 g of carbohydrates and 10 g of fiber can be calculated as if it contains 20 g of carbohydrates. |  |  |
| **Advanced CC knowledge**  **questions** | The carbohydrate-to-insulin ratio is not always constant; it can vary throughout the day. |  |  |
|  | A patient using rapid-acting insulin with a total daily insulin dose of 15 units needs to use 1 unit of insulin for every 30 grams (450/15) of carbohydrates consumed. |  |  |
|  | In the calculation of insulin sensitivity factor, the formula 1500/total insulin dose is used in patients using short-acting insulin |  |  |
